# New Insights on the Burstein-Moss Shift and Band Gap Narrowing in Indium-Doped Zinc Oxide Thin Films

**DOI:** 10.1371/journal.pone.0141180

**Published:** 2015-10-30

**Authors:** K. G. Saw, N. M. Aznan, F. K. Yam, S. S. Ng, S. Y. Pung

**Affiliations:** 1 Physics Programme, School of Distance Education, Universiti Sains Malaysia, 11800, Penang, Malaysia; 2 School of Physics, Universiti Sains Malaysia, 11800, Penang, Malaysia; 3 Institute of Nano-optoelectronics Research and Technology, Sains@USM, 11900, Penang, Malaysia; 4 School of Materials and Mineral Resources Eng., Universiti Sains Malaysia, 14300, Nibong, Tebal, Malaysia; Institute for Materials Science, GERMANY

## Abstract

The Burstein-Moss shift and band gap narrowing of sputtered indium-doped zinc oxide (IZO) thin films are investigated as a function of carrier concentrations. The optical band gap shifts below the carrier concentration of 5.61 × 10^19^ cm^-3^ are well-described by the Burstein-Moss model. For carrier concentrations higher than 8.71 × 10^19^ cm^-3^ the shift decreases, indicating that band gap narrowing mechanisms are increasingly significant and are competing with the Burstein-Moss effect. The incorporation of In causes the resistivity to decrease three orders of magnitude. As the mean-free path of carriers is less than the crystallite size, the resistivity is probably affected by ionized impurities as well as defect scattering mechanisms, but not grain boundary scattering. The *c* lattice constant as well as film stress is observed to increase in stages with increasing carrier concentration. The asymmetric XPS Zn 2p_3/2_ peak in the film with the highest carrier concentration of 7.02 × 10^20^ cm^-3^ suggests the presence of stacking defects in the ZnO lattice. The Raman peak at 274 cm^-1^ is attributed to lattice defects introduced by In dopants.

## Introduction

One of the earliest studies on the optical band gap of degenerate semiconductors showed that the energy gap of InSb widened when metal impurities were present but narrowed as the impurities were removed [1]. For *n*-type degenerate semiconductors, the phenomenon of optical band gap widening was later shown to be related to the filling of the lowest states of the conduction band of its host material [2]. The metal impurity is regarded as a donor, and in a degenerate sample, it introduces a state above the conduction band minimum. In degenerate doping, the Fermi level moves into the conduction band and the height of the Fermi level above the conduction band minimum increases rapidly with increasing carrier concentration [2]. Only optical transitions that involve photon energies that are higher than the band gap of the undoped semiconductor and that are able to make transitions from the valence band up to the corresponding state in the conduction band are allowed, resulting in a band gap widening or the Burstein-Moss shift. As the free carrier concentration increases, the band gap widening is counteracted by a narrowing caused by the correlated motion of charged carriers and by their scattering against ionized impurities [3]. In certain degenerate semiconductors such as InSb the conduction band density of states and the degeneracy concentration are small resulting in a band gap widening for relatively low free carrier concentrations [2]. In other degenerate semiconductors such as ZnO, optical band gap widening is expected to happen for higher carrier concentrations [2].

In recent years, thin films of transparent conducting oxides (TCO) from degenerate semiconductors that have a large optical gap with low electrical resistivity have attracted keen interest due to their potential applications in optoelectronic devices such as light emitting diodes and solar cells [4–6]. Efforts are continuously being made to achieve high carrier concentration and mobility as well as transmittance to improve the performance of transparent electronics [7–8]. Zinc oxide thin film is a promising alternative material for the commonly used tin-doped indium oxide (ITO) because of its low cost, non-toxic nature, abundant availability of zinc and high durability against hydrogen plasma compared with ITO [4–5]. As-grown ZnO is typically an *n*-type semiconductor with a wide direct band gap between 3.2 to 3.4 eV and a high exciton binding energy of 60 meV [9]. In addition, the optical and electrical properties of ZnO films vary with the substrate and annealing temperatures as well as to the addition of metal impurites, creating new applications of the material [10–11]. Being a degenerate semiconductor, the semiconductor-metal transition of ZnO depends on the amount of metal dopants, and films exhibiting metal-like conductivity may result from just adding a small weight percentage of metal impurities. The study on the Burstein-Moss shift and band gap narrowing effect of ZnO-based TCO have thus attracted keen research interest in recent years but certain aspects such as the critical values of the carrier concentrations for band gap widening or the semiconductor—metal transition remain inconclusive. Roth et al. [12–13] reported that for carrier concentrations from 6 × 10^18^ to below 3 × 10^19^ cm^-3^, the optical band gap widening or shift as a function of carrier concentration follows the Burstein-Moss theory of band filling. The onset of gap narrowing, however, is believed to occur at a carrier concentration between 2 × 10^19^ and 3 × 10^19^ cm^-3^ [13]. A higher range of carrier concentration is observed by Lu et al. [14] at the range from 6 × 10^18^ to 4.2× 10^19^ cm^-3^ with the onset of band gap narrowing occurring between 5.4 × 10^19^ and 8.4 × 10^19^ cm^-3^. It should be noted that apart from the critical values of carrier concentrations for the onset of the Burstein-Moss shift and band gap narrowing, previous studies have shown that the structural and electrical properties of the doped ZnO films also vary widely as the carrier concentration increases [15–18].

ZnO films doped with In, a trivalent metal, are potential electrode materials for organic photovoltaic devices [19] and are also better substitutes for SnO_2_ as front electrodes in amorphous Si solar cells due to the possibility of a low temperature deposition as well as stability during hydrogen plasma and high temperature processes [5]. In the present study, the optical band gap as well as the accompanying changes in the lattice constants, resistivity, stress, mean-free path of carriers and crystallite sizes of the In-doped ZnO (IZO) films are investigated as a function of carrier concentration to gain an insight on the Burstein-Moss effect and band gap narrowing of this promising material.

## Materials and Methods

The IZO thin films were fabricated by the r.f. magnetron sputtering method using high purity targets obtained from ACI Alloys at the substrate temperature of 150°C on glass and Si (100) substrates. The targets consist of ZnO and In_2_O_3_. The substrates were cleaned ultrasonically using acetone and methanol and subsequently rinsed in deionized water in the ultrasonic bath. The substrates were finally dried using pure nitrogen gas before being inserted into the sputtering chamber. IZO films with different carrier concentrations are obtained by using targets with different weight percentages of In_2_O_3_ as well as by varying the deposition parameters. The films were sputtered in an argon atmosphere to obtain a thickness of approximately 500 nm.

The optical measurements were performed using a UV-visible dual beam spectrophotometer. For direct transition films such as ZnO, the optical band gap was determined by plotting (*αhv*)^2^ against the photon energy *hv*. The direct band gap was obtained by extrapolating the linear portion of (*αhv*)^2^ and intersecting the photon energy axis, a technique that is sometimes referred to as the Tauc plot. The optical band gap of the as-sputtered (undoped) ZnO film is 3.29 eV, which is consistent with the reported values between 3.28–3.29 eV for as-grown ZnO films [20–21]. The band gap shifts of the IZO samples were calculated using 3.29 eV as the reference value for the undoped ZnO film. The XRD 2-theta scans were obtained using the high resolution PANalytical system with a Cu Kα radiation source (λ = 0.154 nm) operated at 40 kV and 30 mA. During the measurements, data were obtained using 0.05° steps. Changes in lattice parameters, stress and crystallite size were also obtained from the XRD data. Raman measurements were performed using the Ar^+^ ion laser (λ = 514.5 nm) as the excitation source in the backscattering configuration with the Jovin Horiba spectrometer that was equipped with a charged coupled device (CCD) detector and a confocal microscope.

Chemical states of In, Zn and O were investigated using x-ray photoelectron spectroscopy (XPS). High resolution XPS measurements were performed by the PHI Quantera II spectrometer using a monochromated Al Kα radiation (*hν* = 1486.6 eV) as the excitation source. Wide survey spectra and high resolution regional scans of Zn, O and In were acquired using analyzer pass energies of 280 eV (for maximum sensitivity) and 112 eV, respectively. In addition, depth profiling was also performed to study the distribution of In in the ZnO lattice. Pass energy of 280 eV is used for the depth profiling measurements. The films were sputter cleaned using Ar^+^ ion bombardment to reduce ambient contamination. The C 1s peak of adventitious carbon at 284.75 eV was used for charge referencing. A Shirley background subtraction was used for the analysis of core level peaks while a mixed Gaussian and Lorentzian function was used to fit the core level peaks. Resistivity, carrier concentration and mean-free path of carriers were determined from Hall measurements (HL 5500 PC system). The magnetic field in the Hall measurements is 0.32 T. All characterization measurements were done at room temperature.

## Results and Discussion

**Fig 1:**
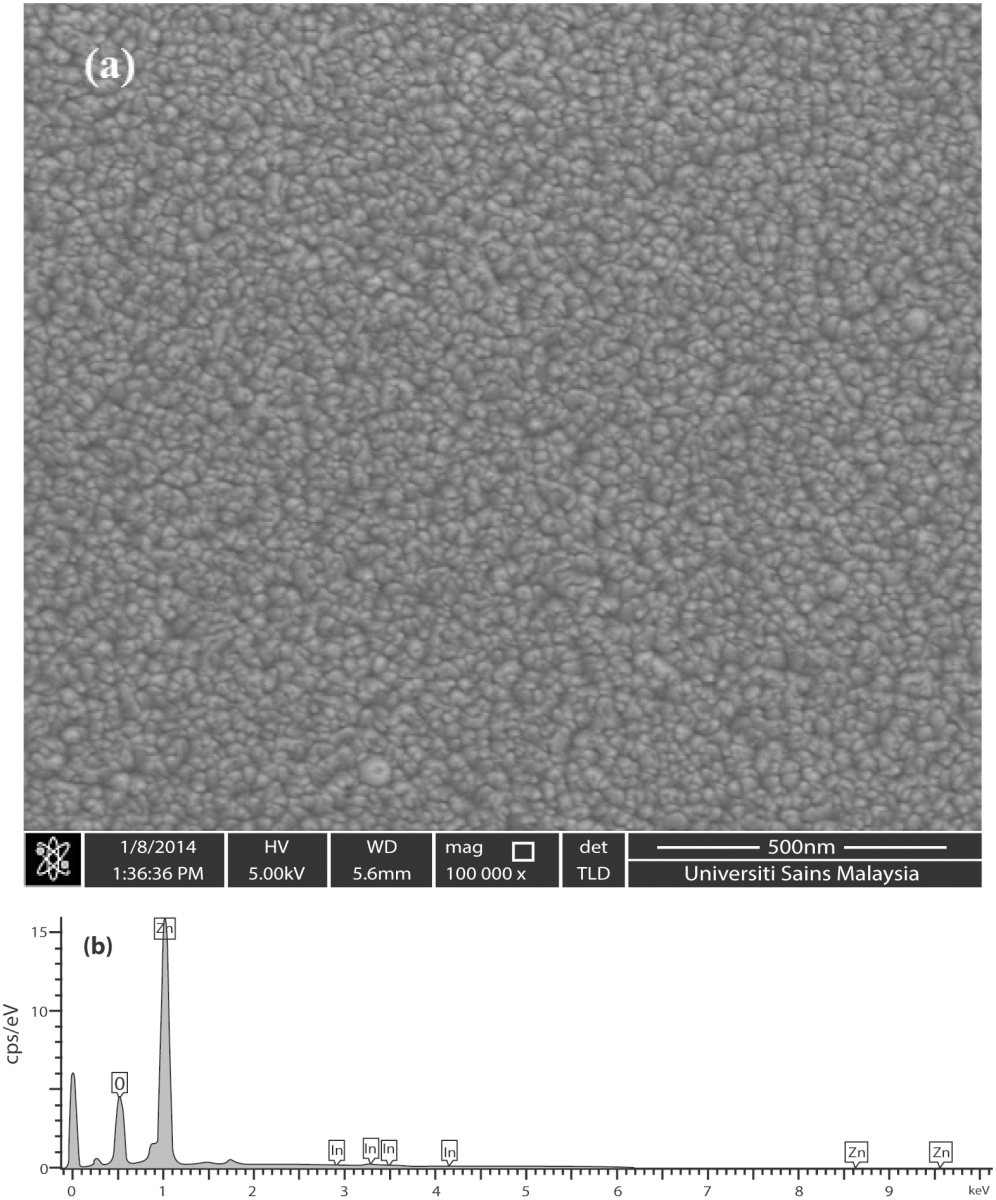
(a) Surface morphology the IZO film; (b) EDS spectrum of the IZO film.

A total of 10 films with different carrier concentrations were fabricated for this study including the undoped sample. Hall measurements indicate that the undoped ZnO film as well as the IZO films exhibit *n*-type conductivity. The films have a continuous surface and do not delaminate from the underlying substrate. No cracks or holes are found in the films in the SEM analysis (Fig 1a). The EDS analysis confirms the presence of In in the ZnO film (Fig 1b). No other metallic impurities are detected.

**Fig 2:**
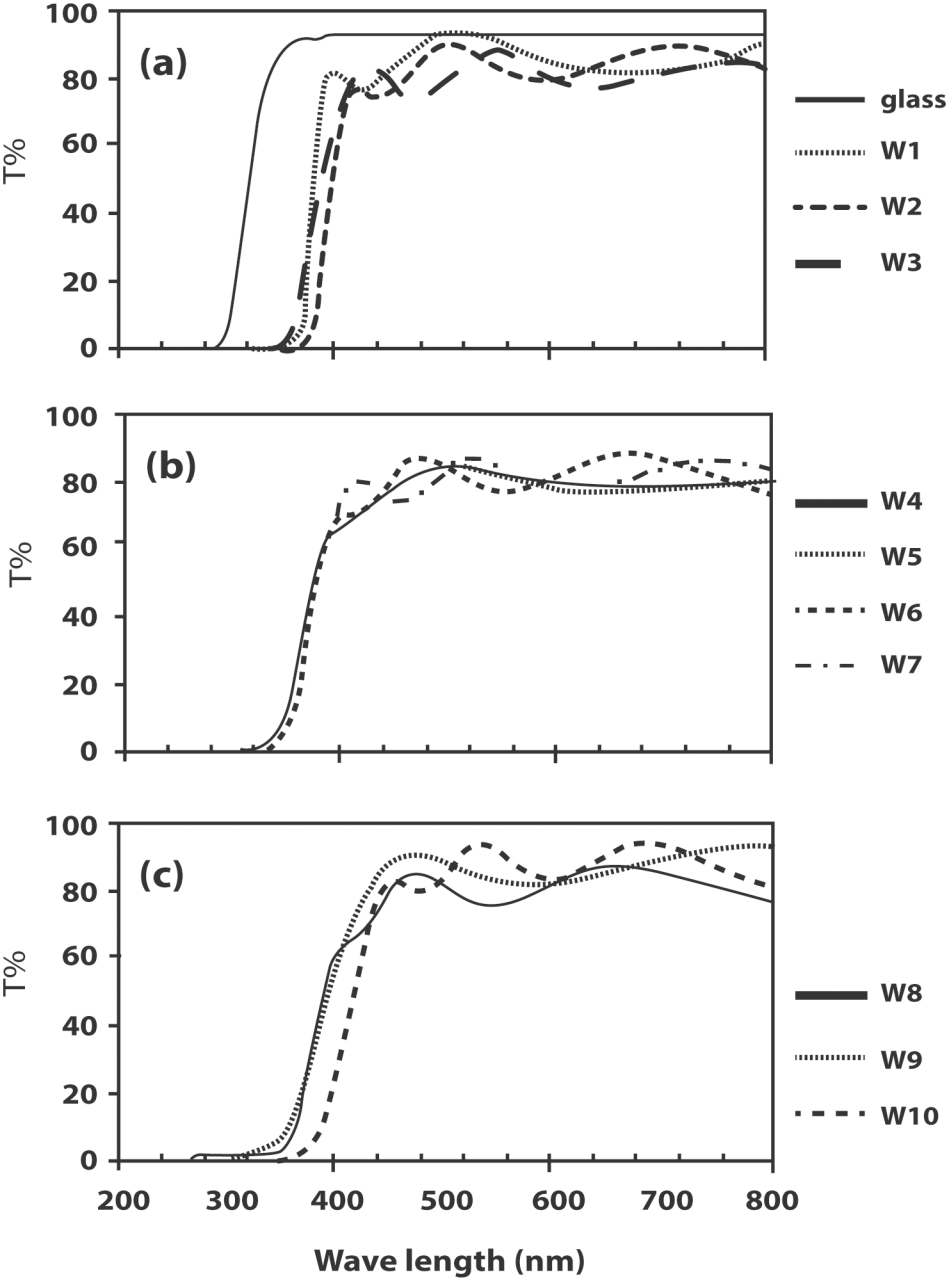
Transmittance of (a) the blank glass, undoped ZnO film (W1) and IZO films W2, W3; (b) IZO films W4 –W7; (c) IZO films W8 –W10.

The inclusion of In in ZnO does not seem to affect the transmittance of the films, which remains generally higher than 80% in the visible region. Fig 2 shows the transmittance of IZO films as well as the transmittance for the undoped ZnO (W1) and blank glass for comparison. For clarity, the transmittance spectra have been divided into Fig 2a–2c.

Table 1 summarizes the optical band gaps and the carrier concentrations of the 10 films. The incorporation of In into ZnO causes the In atom to be ionized into In^3+^ which then replaces the Zn^2+^ ion in the ZnO host lattice. This replacement contributes one free electron and thus increases the carrier concentration. The ionic radii of In and Zn are 0.080 and 0.074 nm, respectively. Thus the incorporation of In into the ZnO host lattice is also expected to cause film stress. The In atom may also reside in the interstitial position and becomes a neutral defect that does not contribute to the free carrier concentration. Another possibility is that the In interstitials may act as donors and contribute to carrier concentration according to the equation

$${\text{In}}^{\text{0}}\to {\text{In}}_{}^{\text{3+}}-3e^{-}$$ (1)

**Table 1:** Variation of optical band gaps with carrier concentrations of IZO films. (Note that W1 is the undoped ZnO film).

Sample	Optical gap (eV)	Carrier concentration (cm^-3^)
W1	3.29	2.56 × 10^18^
W2	3.24	6.92 × 10^18^
W3	3.38	1.66 × 10^19^
W4	3.42	3.58 × 10^19^
W5	3.45	5.61 × 10^19^
W6	3.44	7.64 × 10^19^
W7	3.48	8.71 × 10^19^
W8	3.42	1.94 × 10^20^
W9	3.40	2.63 × 10^20^
W10	3.28	7.02 × 10^20^

In the band filling model, the lowest states in the conduction band are filled with electrons when the carrier concentration increases more than the conduction band density of states, *N*_C_, which can be expressed as

$$N_{C}=2{\left(2\pi m^{*}kT\right)}^{3/2}/h^{3}$$ (2)

**Fig 3:**
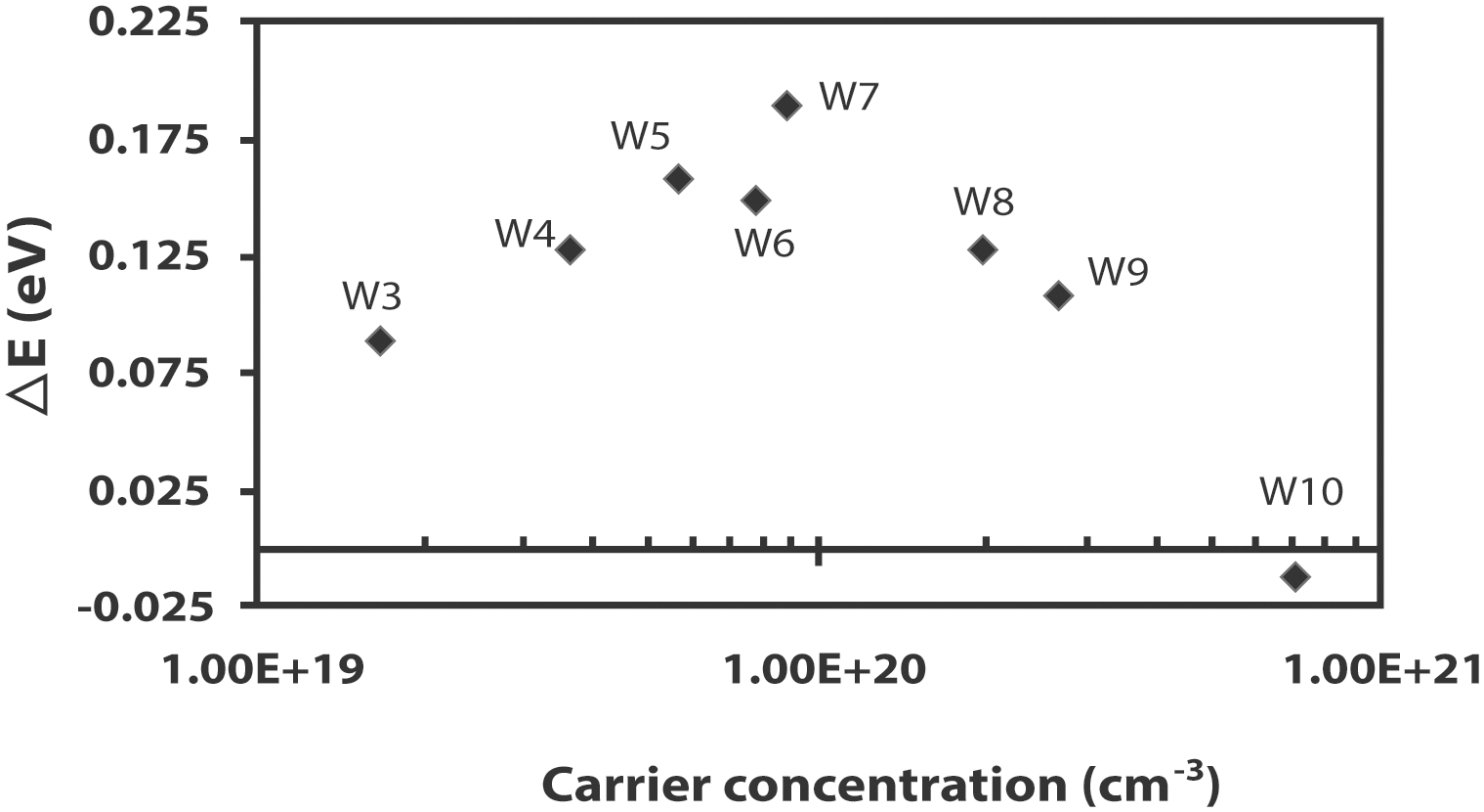
The optical band gap shift of the W3 –W10 films as a function of carrier concentration.

The value of *N*_*C*_ is dependent on the effective mass of electron. An effective electron mass of 0.38 *m*_*0*_ will result in *N*_*C*_ having a value of approximately 6 × 10^18^ cm^-3^. This implies that the optical band gap should increase for the carrier concentrations larger than 6 × 10^18^ cm^-3^. It is interesting to note that films (W3 –W9) with carrier concentrations higher than 6.92 × 10^18^ cm^-3^ exhibit gaps that are higher than 3.29 eV, the optical band gap of the undoped ZnO film (Fig 3). The optical band gap shift has an increasing trend in the range of 1.66 × 10^19^–8.71 × 10^19^ cm^-3^. Beyond the carrier concentration of 8.71 × 10^19^ cm^-3^ the shift decreases although the optical band gaps of W8 and W9 remain higher than that of the undoped ZnO film. Only the optical band gap of W10 is lower than 3.29 eV. The optical band gap shifts of the W3 –W10 films are shown in Fig 3.

In the range where the widening of the optical band gap occurs due to the Burstein-Moss effect the measured optical band gap, *E*_*m*_, is the sum of the optical gap of the as-grown material, *E*_*0*_, plus that due to the filling of the conduction band caused by donors, *ΔE*_*BM*_ [13]. Thus

$$E_{m}=E_{0}+\Delta E_{BM}$$ (3)

In ZnO where a parabolic band is assumed, the band gap energy shift (Δ*E*_*BM*_) due to the Burstein-Moss effect is related to the carrier concentration, *n*, according to the following equation

$$\Delta E_{BM}=\left(h^{2}/8m^{*}\right){\left(3n/\pi \right)}^{2/3}$$ (4)

where *h* is Planck’s constant and m* is the effective mass of the electron [12]. The increase in the optical band gap with increasing carrier concentration is related to the rise of the Fermi level in the conduction band of a degenerate semiconductor.

**Fig 4:**
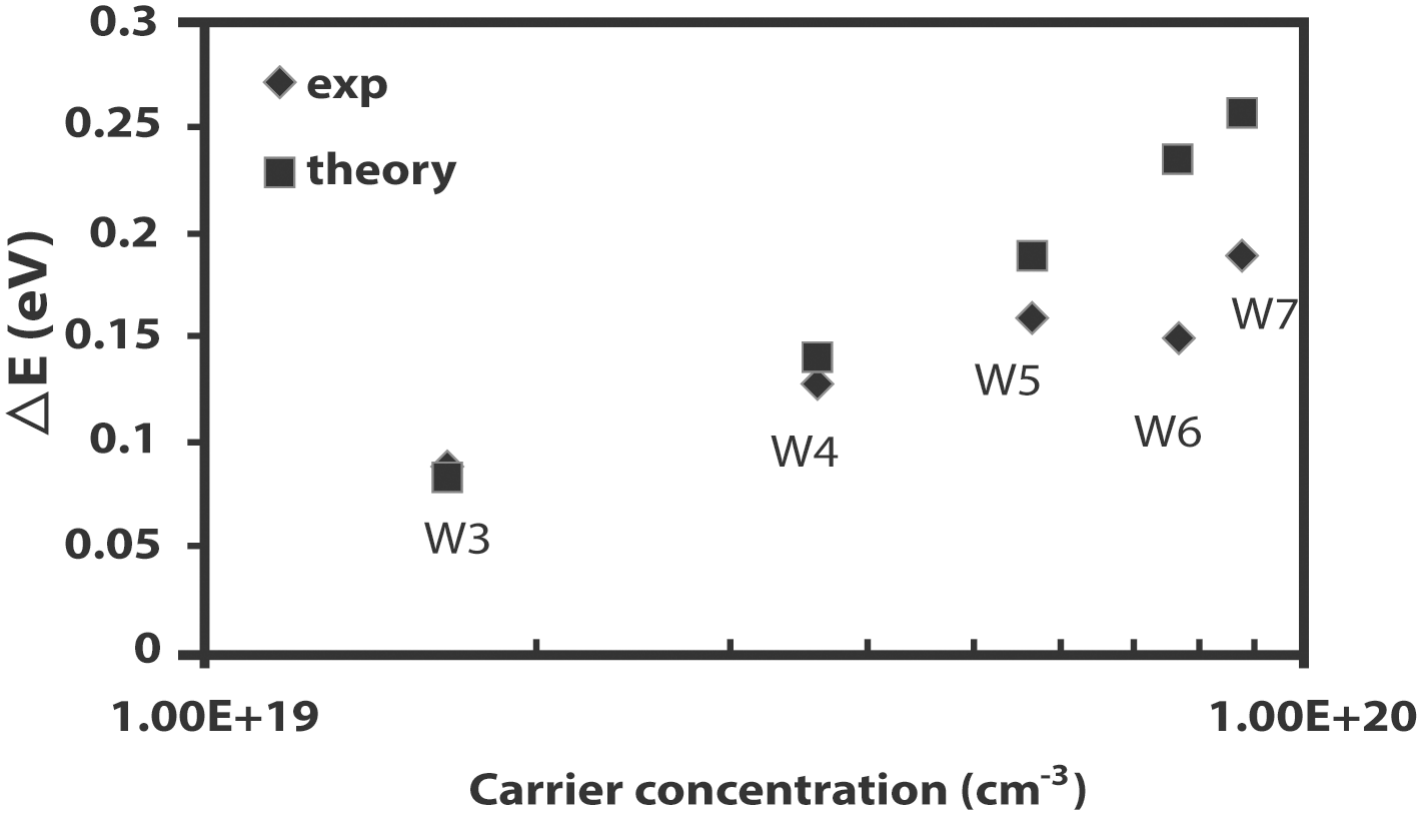
Comparison of the Burstein-Moss shift as a function of carrier concentration obtained by experiment as well as by theoretical calculation.

A comparison between the band gap energy shift obtained from experimental data and theoretical prediction of the Burstein-Moss shift in Fig 4 indicates that the optical band gap shifts below the carrier concentration of 5.61 × 10^19^ cm^-3^ are well-described by the Burstein-Moss effect. The magnitude of the measured optical band gaps, however, is less than the theoretical shift expected for films with higher carrier concentrations. It is believed that the nature and strength of the interaction potentials between donors and the ZnO host results in band gap shrinkage or narrowing [12]. Band gap narrowing, ΔE_g_, is deduced as the difference between the expected Burstein-Moss shift and the measured optical band gap [12]. In Fig 4, a difference in ΔE_g_, (~ 0.03 eV) in W5 can be interpreted as evidence of band gap narrowing. The onset of band gap narrowing is likely to occur between the carrier concentration of W4 (3.58 × 10^19^ cm^-3^) and that of W5 (5.61 × 10^19^ cm^-3^). The critical value of carrier concentration for the onset of band gap narrowing is within the range of carrier concentration reported by Lu et al. [14] as well as Kim and Park [22] but is slightly higher than the range suggested in an earlier study by Roth et al. [13].

The smaller measured optical band gap shifts observed in films with higher carrier concentrations indicate that as the carrier concentration increases the band gap narrowing mechanisms become increasingly significant and are competing with the Burstein-Moss effect. It has been previously reported that band gap narrowing effect should not be neglected for carrier concentrations more than 10^19^ cm^-3^ [23]. The carrier concentration at which band gap narrowing and the related semiconductor-metal transition are expected to occur in ZnO (the so-called Mott critical concentration, *n*_*c*_) can be determined by the relation

$$n_{c}^{1/3}a^{*}=K$$ (5)

where a* is the Bohr radius of the donor in Å and *K* is a constant that varies from 0.18 to 0.376 [14]. In the effective-mass approximation the donor radius is

$$a^{*}=\left(\frac{\varepsilon }{m^{*}}\right)\left(\frac{\hbar^{2}}{e^{2}}\right)$$ (6)

Using *ε* = 8.65*ε*_*0*_ as the dielectric constant of ZnO and m* = 0.28*m*_0_ (*m*_*0*_ is the mass of electron), the donor radius is 14.73Å. Based on the range of *K* values, *n*_*c*_ is found to vary from 1.8 × 10^18^ to 1.6 × 10^19^ cm^-3^. The onset of band gap narrowing in this present study occurs between the carrier concentration of 3.58 × 10^19^ and 5.61 × 10^19^ cm^-3^ and is therefore within the accepted theoretical range of *n*_*c*_ to 10 *n*_*c*_ [21]. A corresponding decrease in resistivity should also be observed.

**Fig 5:**
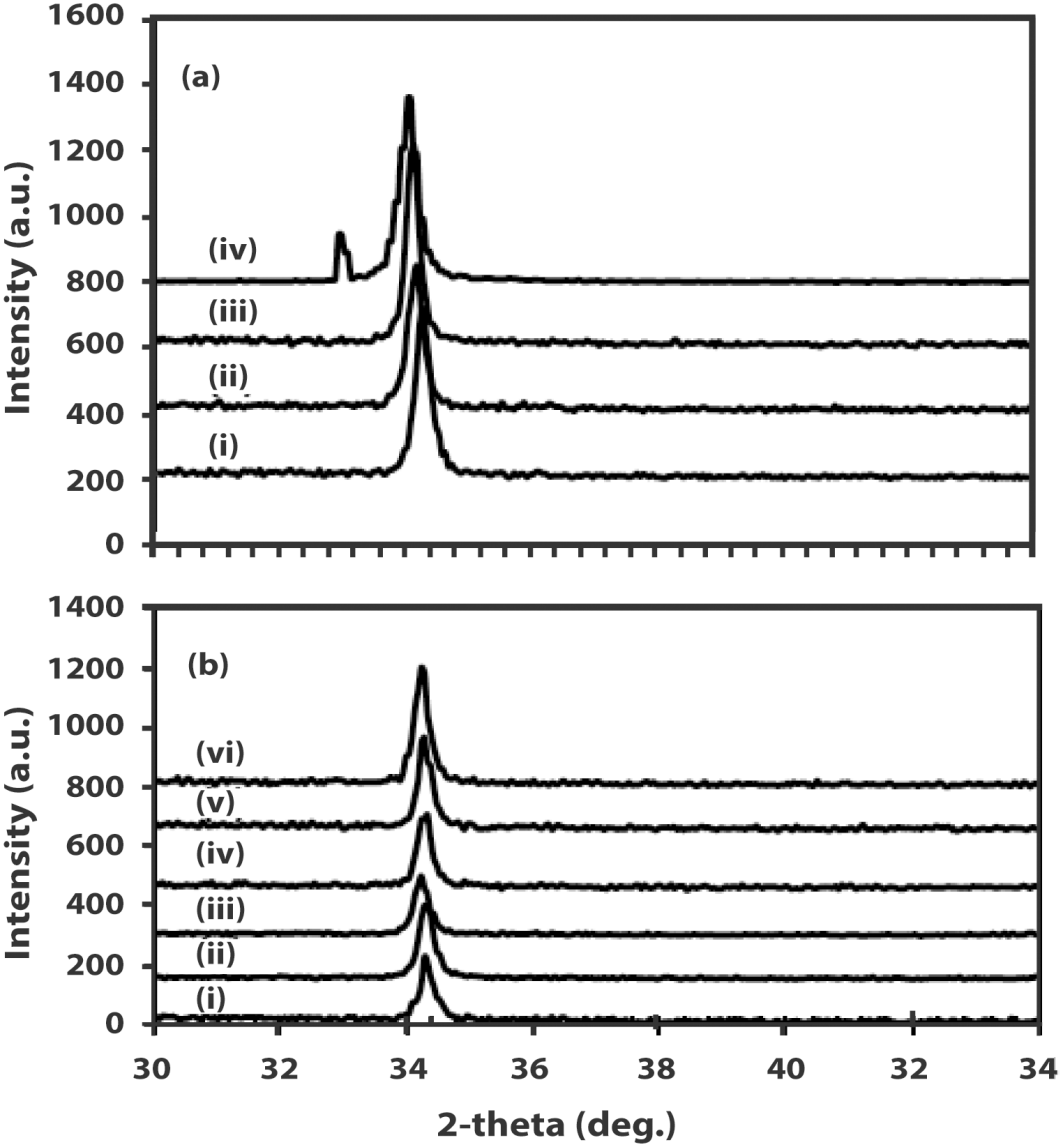
XRD 2-theta spectra of (a) (i) W7 (ii) W8 (iii) W9 (iv) W10; (b) (i) W1 (ii) W2 (iii) W3 (iv) W4 (v) W5 (vi) W6.

The undoped ZnO as well as the IZO films have a preferential (002) growth direction with the *c*-axis perpendicular to the substrate surface. A distinct and intense ZnO (002) peak indicates a hexagonal wurtzite structure. This implies that IZO films maintain the hexagonal wurtzite structure of ZnO despite the incorporation of In within the host lattice. No diffraction peaks that belong to In_2_O_3_ are observed in all the samples. The undoped ZnO thin film exhibits a (002) ZnO peak position at 2*θ* = 34.3144° with a FWHM value of 0.1476°. The ZnO (002) peak positions of the IZO films are lower than that of the undoped ZnO. There is a general decrease in the value of 2*θ* from the value of the undoped ZnO film as well as the FWHM values as the carrier concentration increases. The (002) peak of W10, which has the highest carrier concentration, is observed at 2*θ* = 34.0354° with a FWHM value of 0.3936°. The shift of the (002) peak to lower 2*θ* values and the higher FWHM values for the IZO films suggest that there is an increase in film stress due to In incorporation. It should be noted that although the ZnO wurtzite structure may be preserved in Zn-rich IZO films, increasing In content induces structural stacking defects, perturbing the Zn ion array [24]. Sample W10 exhibits an additional peak at 2*θ* = 33.0160° which is the Si (200) peak originating from the underlying Si substrate. Fig 5 shows the XRD patterns of all the films while Table 2 summarizes the ZnO (002) peak positions as well as the full-width-half-maxiumum values.

**Table 2:** ZnO (002) peak positions with the FWHM values of W1- W10.

Sample	2-theta (deg)	FWHM (deg)
W1	34.3144	0.1476
W2	34.2987	0.2460
W3	34.2173	0.1968
W4	34.2744	0.2460
W5	34.2753	0.2400
W6	34.2193	0.2400
W7	34.2699	0.1968
W8	34.1457	0.2460
W9	34.1047	0.1968
W10	34.0354	0.3936

**Fig 6:**
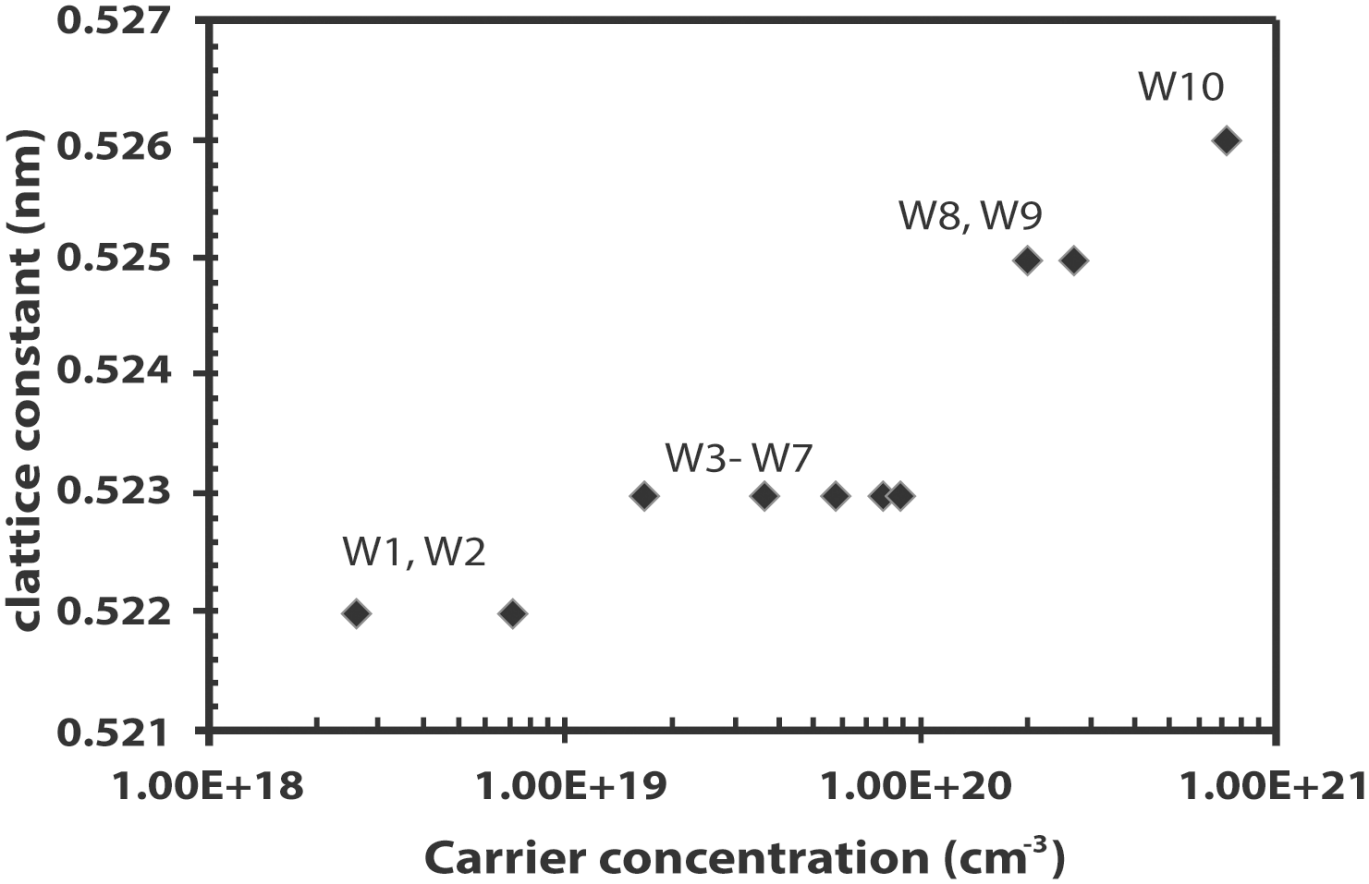
Variation of the *c*-lattice constant with carrier concentration.

The lattice constant of ZnO, *c*, is determined from the (002) peak using the equation for hexagonal lattice as shown below

$$c=\frac{\lambda }{\text{sin }\theta }$$ (7)

where λ is the wavelength of the x-ray (0.154 nm) and *θ* is the Bragg angle. Fig 6 shows that the elongation of the *c*-lattice constant as a result of In incorporation occurs in steps of 0.001 nm when the *c*-lattice constant is calculated to the nearest three decimal points. The samples show four sets of *c*-lattice constants apparently based on the distinct stages of carrier concentration. Samples W1 (undoped ZnO) and W2, which can be categorized as pre-Burstein-Moss shift, possess a lattice constant of 0.522 nm while samples that exhibit the Burstein-Moss shift (W3 –W7) show a lattice constant value of 0.523 nm. The Burstein-Moss effect appears to cause the *c*-lattice constant to elongate by 0.001 nm. It is interesting to note that while samples W3 to W7 show increasing carrier concentration, the *c*-lattice constant remains at 0.523 nm. The next stage of elongation, however, is observed at the range of carrier concentrations associated with a sudden decrease in the optical band gap. The lattice constant of samples in this range (W8 and W9) is observed to elongate a further 0.002 nm to 0.525 nm. Sample W10 with the highest carrier concentration and the most amount of incorporated In has the largest lattice constant of 0.526 nm. It is likely that the lattice elongation does not occur continuously but in stages as In is incorporated in the ZnO lattice.

The elongation of the lattice constant is generally attributed to the presence of defects due to interstitials. A recent study reported that an increase in lattice constants of ZnO after Co doping is due to Co interstitials [25]. Evidence of lattice constant expansion due to interstitials has also been reported in studies that involve the incorporation of B as a dopant into chemical vapor deposition (CVD) diamond thin films [26–27].

**Fig 7:**
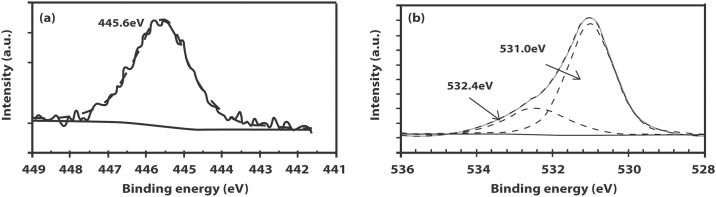
XPS spectra of the (a) In 3d_5/2_ peak (b) O 1s peak.

**Fig 8:**
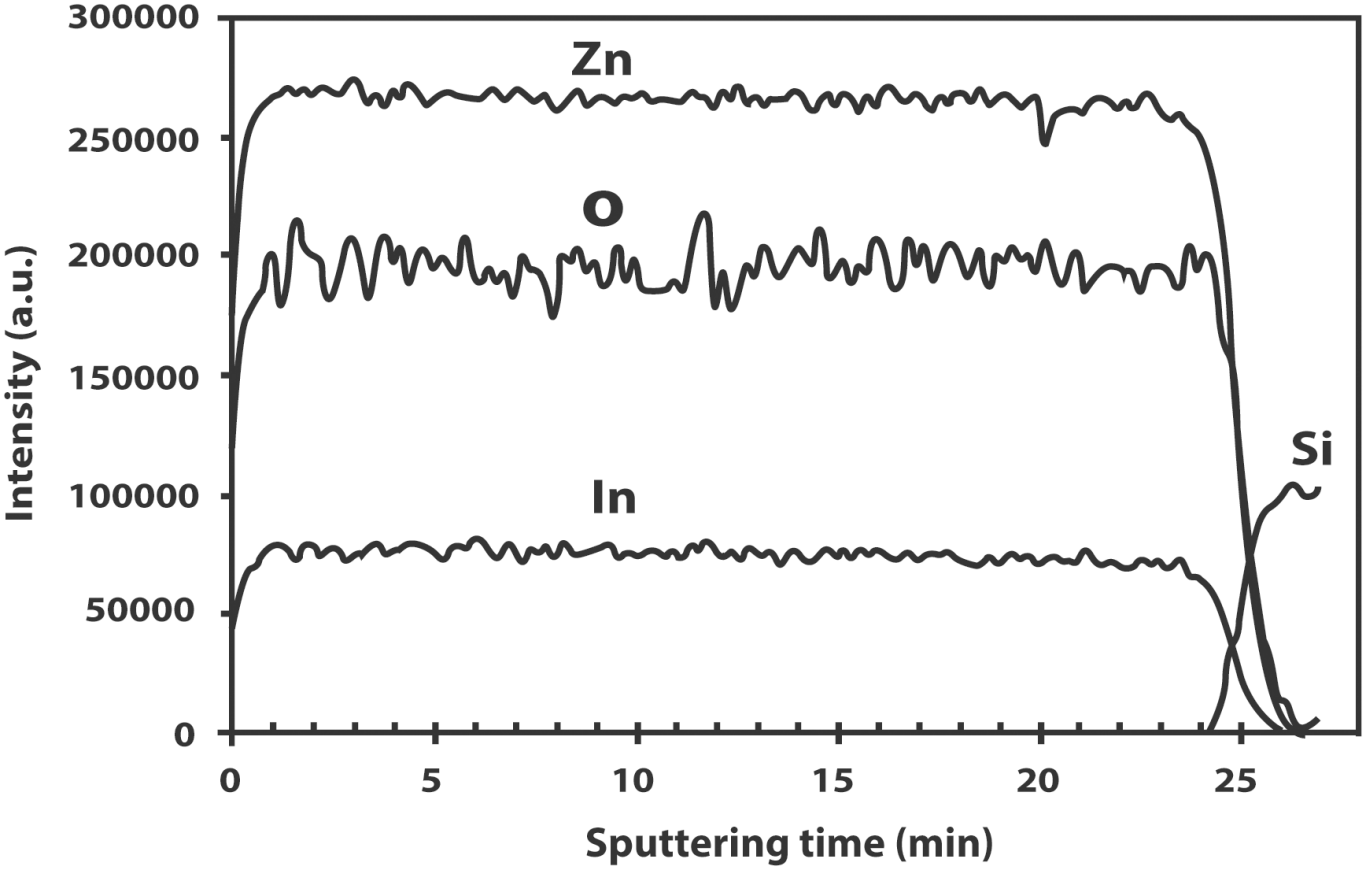
XPS depth profiling spectrum of IZO film on Si substrate.

The XPS wide survey scans indicate that the IZO films only exhibit peaks belonging to In, Zn, O and adventitious carbon peaks. The In 3d_5/2_ spectra can be resolved into single component peaks with binding energies lying between 444.4–445.6 eV, which are within the reported values for films prepared by r.f. magnetron sputtering (444.5 eV) as well as by spray pyrolysis (445.1 eV) [28–29] but are higher than the binding energies reported for pure metallic In foil (443.4–443.8 eV) [30]. The higher binding energy of the In 3d_5/2_ peaks is attributed to In residing in the ZnO matrix and forming an unknown complex [29]. The O 1s peak can be deconvoluted into two components. The component with a low binding energy at approximately 531.0 eV can be attributed to the O^2−^ ions of ZnO in the wurtzite structure of hexagonal Zn^2+^ ion array while the higher binding energy component at approximately 532 eV is due to oxygen belonging to surface adsorbed species such as CO_3_, adsorbed OH or adsorbed oxygen [31]. Fig 7a shows the W7 In 3d_5/2_ peak at 445.6 eV while Fig 7b shows the components of the O 1s peak at binding energies 531.0 and 532.4 eV, respectively. The depth profiling measurements indicate that In is distributed evenly throughout the thickness of the ZnO film and not just at the surface of the film (Fig 8).

**Fig 9:**
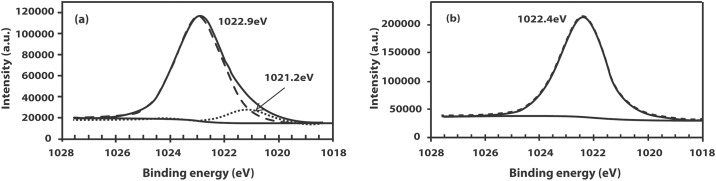
The Zn 2p_3/2_ peak of (a) W10 (b) W7.

Fig 9a reveals the asymmetric Zn 2p_3/2_ peak of W10 from two resolved peaks at binding energies of 1022.9 and 1021.2 eV. The former peak has a higher intensity and is attributed to Zn in the oxide form while the latter is attributed to elemental Zn. This implies that W10 which is the IZO film with the highest carrier concentration is Zn-rich. No asymmetrical nature, however, is observed for the Zn 2p_3/2_ peak of other IZO films as well as the undoped ZnO film. The Zn 2p_3/2_ peaks of these films are all single component corresponding to the Zn–O bond. Fig 9b shows the Zn 2p_3/2_ peak of W7 at 1022.4 eV. A recent HRTEM study suggests that Zn-rich ZnO-based films (such as IZO films) with a wurtzite structure can develop structural stacking defects with increasing incorporation of metal impurities [24]. Based on these findings the slight shift in binding energy as well as the asymmetric feature of the Zn 2p_3/2_ peak for W10 is attributed to a stacking fault due to the heavy incorporation of In in the ZnO hexagonal wurtzite structure.

**Fig 10:**
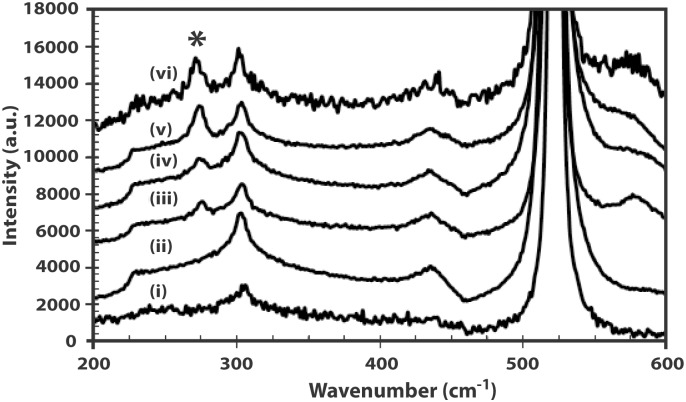
Raman spectra of (i) Si substrate (ii) undoped ZnO (iii) W7 (iv) W8; (v) W9 (vi) W10. The peak at 274 cm^-1^ attributed to lattice defect due to In incorporation is indicated with an asterisk.

Evidence of increasing structural defects in the hexagonal wurtzite lattice with increasing In incorporation can be seen in the deteriorating E_2_(high) peak at 438 cm^-1^ in the Raman measurements for the IZO film with the highest carrier concentration (W10) as compared with the undoped ZnO and other IZO films with lower carrier concentrations (Fig 10). Hexagonal wurtzite ZnO belongs to the space group $C_{6v}^{4}$ and according to group theory, the Raman active zone center optical phonons are A_1_+E_1_+2E_2_+2B_1_. The A_1_ and E_1_ modes are polar modes whereas the E_2_ modes are non-polar. The B_1_ modes are silent modes. The E_2_(high) peak is well-defined in the undoped ZnO film as well as the films with lower carrier concentrations. This implies that the wurtzite structure starts to deteriorate with heavy doping beyond the carrier concentration of 7 × 10^20^ cm^-3^. The incorporation of In into ZnO also introduces a peak at 274 cm^-1^ (indicated with an asterisk in Fig 10) which increases with the carrier concentration of the IZO films. No peak at 274 cm^-1^ is observed for undoped ZnO as it is not associated with the wurtzite structure. The peaks at 303 and 520 cm^-1^ originate from the underlying Si(100) substrate. The anomalous Raman mode has been assigned previously to local vibrational modes of nitrogen [32]. However, the assignment to nitrogen incorporation is doubtful as this peak has been observed in ZnO films doped with Fe, Sb, Al and Ga, and which have been grown intentionally without nitrogen [33]. Since this peak is absent in the undoped ZnO film and as the mass of the dopants differs largely, lattice defects are the likely source of this Raman mode.

**Fig 11:**
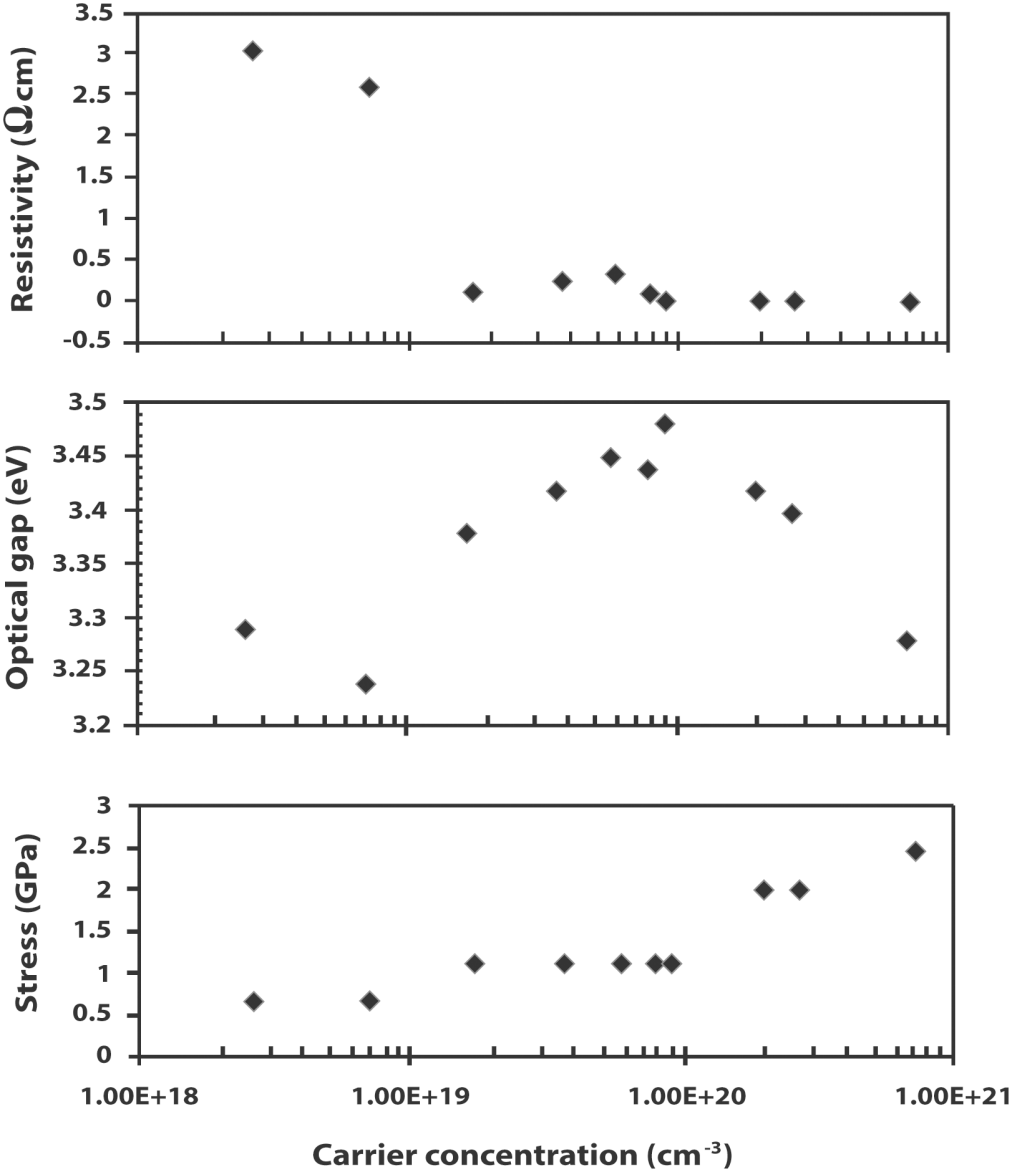
Comparison of changes in resistivity, optical band gap and stress with carrier concentration.

Fig 11 shows the variation of the optical band gap with the resistivity and stress of the films. The resistivity decreases abruptly when the ZnO films are doped with In. The resistivity of the undoped ZnO is 3.037 Ω cm while the lowest resistivity of the IZO films is 3 × 10^−3^ Ω cm. The incorporation of In releases free electrons causes a decrease in resistivity of about three orders of magnitude. Fig 11 also shows an immediate decrease in resistivity as In is introduced into ZnO. There is a further decrease in resistivity from 2.51 × 10^−1^ Ω cm to 10^−3^ Ω cm that corresponds to the onset of band gap narrowing. The films with the lowest resistivity (W8 –W10) thus show metal-like conductivity. The lowest resistivity for films prepared using sol-gel technique as well as chemical vapor deposition reported in previous studies is 5.54 × 10^−1^ Ω cm and 4.5 × 10^−1^ Ω cm, respectively [16, 34]. It should be noted that the latter exhibits a resistivity that decreases with increasing doping from 7 × 10^−2^ to about 4.5 × 10^−3^ Ω cm before increasing again to approximately 10^−2^ Ω cm [16].

The stress parallel to the film surface is derived from X-ray measurements [35] using the following equation

σ=2c132−c33(c11+c12)2c13×c−coco (8)

The elastic constants *c*_ij_ are based on data from single crystalline ZnO where *c*_11_ = 208.8, c_33_ = 213.8, *c*_12_ = 119.7 and *c*_13_ = 104.2 GPa. Thus the film stress can be expressed simply as

$$\sigma =-233\times 10^{9}\left(\frac{c-c_{o}}{c_{o}}\right)$$ (9)

where *c* is the lattice constant of the film and *c*_*o*_ is the standard value taken to be 0.5205 nm (JCPDS 79–2205). From the XRD analysis, it is found that the IZO films experience an increase in the amount of stress as the carrier concentration increases. The increase in stress from– 0.671 GPa (for the undoped ZnO film) to– 2.462 GPa is attributed to the incorporation of In in the ZnO host lattice. The negative values indicate the existence of tensile stress in the films, which is consistent with the elongation of the *c*-axis lattice parameter. Since the film stress is derived from the XRD measurements, its pattern of increase is similar to that observed for the elongation of the c-lattice constant. The film stress, for instance, remains constant during the Burstein-Moss shift (W3 –W7) as well as during the occurrence of band gap decrease (W8 –W9).

**Fig 12:**
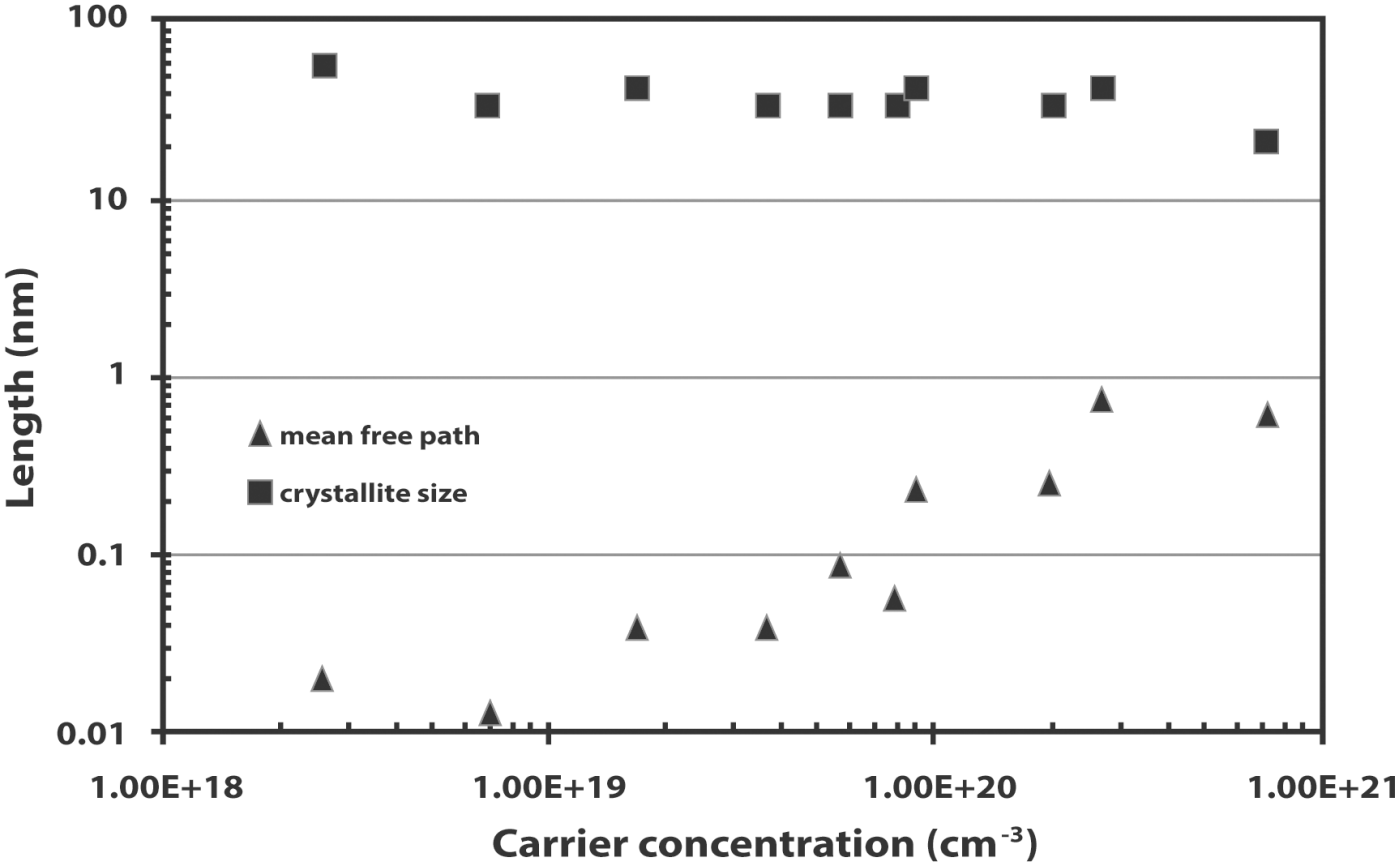
Comparison of the mean-free path of the carrier and crystallite size of the IZO films with carrier concentration.

Fig 12 shows the comparison of the mean-free path of the carriers and the crystallite sizes of the films. The mean-free path is calculated using [36]

$$l=\left(\frac{h}{2e}\right){\left(\frac{3n}{\pi }\right)}^{1/3}\mu$$ (10)

where *μ* is the mobility, *h* is the Planck’s constant, *e* is the charge of the electron and *n* is the carrier concentration. The mean-free path of the undoped ZnO film is 0.02 nm. The crystallite size is calculated using the Debye Scherrer formula as shown below

$$D=\frac{0.9\lambda }{\beta \text{cos}\theta }$$ (11)

where *D* is diameter of the crystallites, *λ* is the wavelength of Cu Kα line, *β* is the FWHM in radians and *θ* is the Bragg angle. The as-sputtered (undoped) ZnO film has a crystallite size of 56 nm. The crystallite size of the IZO films remains fairly constant in the same order of magnitude while the mean-free path of the carrier appears to be divided into two different orders of magnitude as the carrier concentration increases. The mean-free path increases an order of magnitude for films with carrier concentrations higher than 8.71 × 10^19^ cm^-3^. Longer mean-free paths are thus observed for films with a higher carrier concentrations. Grain boundary scattering will be dominant if the crystallite size is comparable with the carrier mean-free path but since all the samples have a smaller mean-free path than the crystallite size, other scattering mechanisms such as impurity scattering and lattice vibration scattering may be more influential.

## Conclusions

We have investigated the changes of the optical band gap, lattice constant, resistivity, film stress, mean-free path of carriers and crystallite sizes of the IZO films as a function of carrier concentration up to 7.02 × 10^20^ cm^-3^ to gain an insight on the Burstein-Moss effect and band gap narrowing. The optical gap shift of films with carrier concentrations below 5.61 × 10^19^ cm^-3^ seems to be well-described by the Burstein-Moss effect. The onset of band gap narrowing is believed to occur in the range of 3.58 × 10^19^ to 5.61 × 10^19^ cm^-3^, and corresponds to a decrease in resistivity. The incorporation of In causes the resistivity to decrease three orders of magnitude from ~ 3 Ω cm to 10^−3^ Ω cm. Impurity as well as defect scattering mechanisms are believed to be major factors affecting the resistivity as the mean-free path of carriers is less than the crystallite size. The incorporation of In also causes the elongation of the *c*-lattice constant and an increase in film stress. The Raman anomalous local vibrating mode at ~ 274 cm^-1^ is attributed to lattice defects introduced by In dopants. XPS measurements suggest that stacking defects in the ZnO lattice could have occurred in the most heavily doped sample.
